# Etude rétrospective des fractures de Galeazzi chez l’adulte dans le Département d’Orthopédie du Centre Hospitalier Universitaire Habib Bourguiba Sfax, Tunisie: à propos de 45 cas

**DOI:** 10.11604/pamj.2020.35.135.22612

**Published:** 2020-04-21

**Authors:** Nizar Sahnoun, Boubaker Ayedi, Khaled Keskes, Mohamed Ali Rebai, Imen Zouch, Wassim Zribi, Zoubaier Ellouz, Hassib Keskes

**Affiliations:** 1 Service de Chirurgie Orthopédique et Traumatologie, CHU Habib Bourguiba, Sfax, Tunisie

**Keywords:** Fracture luxation de Galeazzi, radius, traitement chirurgical, Galeazzi fracture-dislocation, radius, surgery

## Abstract

La fracture de Galeazzi est définie par l'association d'une fracture du radius et une luxation radio-ulnaire inférieure. Son diagnostic est souvent méconnu initialement. Le but de notre travail est de déterminer l'aspect épidémio-clinique des fractures luxation de Galeazzi chez l'adulte et d'apprécier les résultats fonctionnels et radiologiques de notre série. Il s'agit d'une étude rétrospective descriptive, sur une période allant de 2009 à 2018 colligée au Service d'Orthopédie du CHU Habib Bourguiba Sfax, portant sur 45 cas de fractures de Galeazzi traitées chirurgicalement. Nous avons utilisé le score de Mestdagh pour l'évaluation clinique de nos résultats. Le délai moyen de prise en charge était 5,35 jours. La synthèse du radius a été pratiquée par plaque vissée dans 39 cas et la radio-ulnaire a été embroché dans 13 cas. La consolidation a été obtenue dans un délai moyen de 10,5 semaines. Les résultats étaient excellents chez 35 patients, bon chez 3 patients, moyen chez 6 patients et mauvais chez un patient. Nous avons déploré 3 cas de sepsis sur broche et deux cas de cal vicieux. La fracture luxation de Galeazzi reste une entité sous diagnostiquée dans certains cas. Un examen dynamique peropératoire après synthèse solide du radius a permis d'avoir de bons résultats fonctionnels.

## Abstract (en)

Galeazzi fracture is defined as the association of radius fracture and inferior radioulnar joint dislocation. Its diagnosis is often unrecognized initially. The purpose of our study is to determine the epidemioclinical features of Galeazzi fracture-dislocations in adults and to assess the functional and radiological outcomes of our case series. We conducted a retrospective descriptive study of 45 patients with Galeazzi fractures treated surgically whose data were collected in the Department of Orthopaedics at the Habib Bourguiba University Hospital, Sfax, Tunisia, over a period ranging from 2009 to 2018. We used Mestdagh score for the clinical evaluation of the results. The mean time between fracture and treatment initiation was 5.35 days. Fixation of the radius was performed using screwed plate in 39 cases and fixation of the radio-ulnar joint using a plug in 13 cases. The average time for union was 10.5 weeks. Outcomes were excellent in 35 patients, good in 3 patients, medium in 6 patients and adverse in a patient. We had 3 cases of sepsis at the level of the plug and two cases of malunions. Galeazzi fracture-dislocation is an under diagnosed disorder in some cases. Dynamic intraoperative examination after solid fixation of the radius can allow for good functional results.

## Introduction

La fracture luxation de Galeazzi est définie par l'association d'une fracture du radius et une luxation de l'articulation radio-ulnaire distale (RUD). Elle est initialement décrite par Sir Astley Cooper en 1822 et publiée par Riccardo Galeazzi en 1934 [1]. L'incidence de cette entité chez l'adulte est de 2,7 à 6,8% de l'ensemble des fractures de l'avant-bras [2]. La fracture luxation de Galeazzi pose un problème de diagnostic, en effet, elle est souvent méconnue ou peut passer inaperçue. Elle pose aussi un problème thérapeutique et pronostic vu que cette lésion tire sa gravité surtout de la luxation radio-ulnaire distale qui en constitue l'urgence et dont la méconnaissance retarde le traitement de la lésion ligamentaire ce qui retentit gravement sur la fonction de la prono-supination. Nous présentons dans ce travail une étude rétrospective de 45 cas de fractures de Galeazzi chez des patients traités dans le Service de Chirurgie Orthopédique du Centre Hospitalo-Universitaire Habib Bourguiba de Sfax sur une période de 10 ans allant de 2009 à 2018. L'objectif de notre travail est de déterminer les aspects épidémio-cliniques des fractures luxation de Galeazzi et d'évaluer nos résultats fonctionnels et radiologiques.

## Méthodes

**Figure 1:**
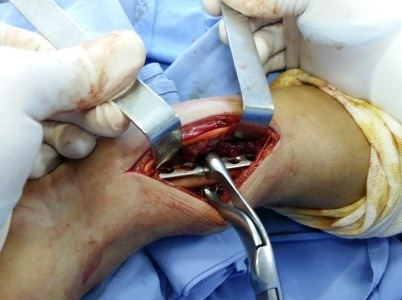
Voie d'abord de Henry

Nous avons mené une étude rétrospective descriptive portant sur tous les cas de fracture luxation de Galeazzi chez les patients hospitalisés au Service d'Orthopédie et Traumatologie du CHU Habib Bourguiba de Sfax entre le 1^er^ janvier 2009 et le 31 décembre 2018. Le recueil des données a concerné les données épidémiologiques: l'âge, sexe, les antécédents personnels, le coté dominant, le coté atteint, les circonstances du traumatisme, le mécanisme, le délai de consultation et les complications immédiates. Le bilan radiologique a comporté une radiographie de l'avant-bras de face et de profil, une radiographie du poignet de face et de profil et une radiographie du coude de face et de profil. Nous avons adopté la classification de Mansat *et al*. pour classer nos fractures. Nous avons noté également le délai de consultation et le délai de prise en charge. La voie d'abord utilisée est la voie antérieure de Henry en cas d'ostéosynthèse interne par plaque vissée (Figure 1). La stabilité de l'articulation radio-ulnaire distale a été déterminée en peropératoire par des manœuvres dynamiques sous contrôle scopique.

Si elle est jugée instable, on a eu recours à un embrochage transversal de l'articulation radio-ulnaire suivi d'une immobilisation plâtrée et lorsqu'elle est jugée stable, on opte pour une immobilisation plâtrée en supination. Le recours au fixateur externe était réalisé dans la fracture ouverte. On a établi un protocole de rééducation qui consiste à lutter contre l'œdème et l'inflammation, le renforcement musculaire contre résistance, la récupération des amplitudes articulaires, la rééducation active visant à récupérer la force musculaire et la prono-supination. Parmi les nombreux critères décrits dans la littérature nous avons retenu celle proposée par l'équipe de Mestdagh pour évaluer nos résultats. Cette cotation tient compte de quatre critères: des données subjectives fournies par l'interrogatoire concernant la douleur, des données objectives fournies par l'examen clinique en ce qui concerne la force de préhension, saillie de la tête ulnaire, la mobilité du poignet et la prono-supination. La sommation de chacun de ces critères varie entre 6 en minimum et 20 en maximal. Le résultat est jugé: très bien: 6-7 points bien: 8-12 points passable: 13-16 points mauvais: 17-20 points. Les critères radiologiques de consolidation sont la formation d'une cal osseuse mâture avec consolidation du foyer radial sans cal vicieux.

## Résultats

**Figure 2:**
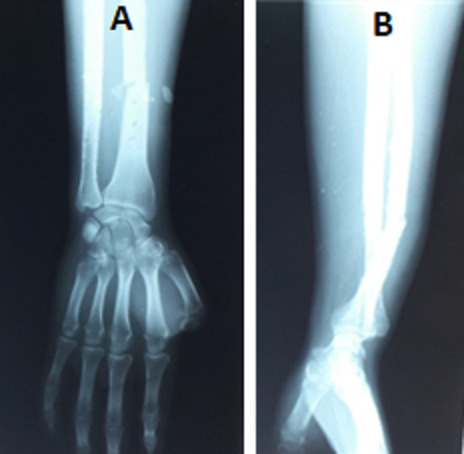
Fracture de Galeazzi stade II de Mansat: (A) face; (B) profil

**Figure 3:**
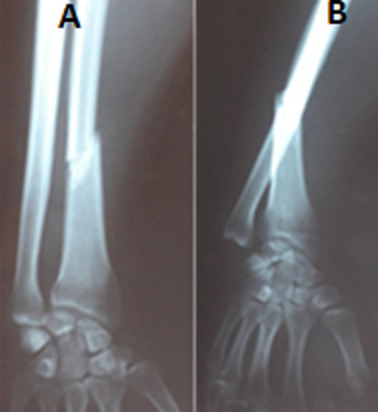
Fracture de Galeazzi stade III de Mansat: (A) face; (B) profil

L'âge moyen de nos patients était 38,5 ans (17-71 ans) avec une prédominance masculine (43 hommes, 2 femmes). L'étiologie prédominante était l'accident de la voie publique dans 23 cas dont 3 polytraumatisés, suivis par les accidents domestiques chez 14 patients et par les agressions chez 4 patients. La fracture était ouverte dans 5 cas. Le côté gauche était atteint dans 24 cas et le côté droit dans 21 cas. Aucun cas bilatéral n'a été noté. L'examen clinique initial a retrouvé une attitude du traumatisé du membre supérieur avec impotence fonctionnelle totale avec un blocage de la prono-supination. Tous les patients n'ont pas présenté de déficit vasculo-nerveux. La fracture était transversale dans 29 cas, oblique dans 11 cas et comminutive dans 5 cas. La luxation de la tête ulnaire était dorsale dans 90% des cas et ventrale dans 10% des cas. Le siège de la fracture était au niveau du tiers distal dans 29 cas et au niveau du tiers moyen dans 16 cas. Selon la classification de Mansat et al les fractures luxations de Galeazzi étaient type I dans 3 cas, type II dans 17 cas (Figure 2) et de type III dans 23 cas. 2 cas de type IV a été noté (Figure 3).

**Figure 4:**
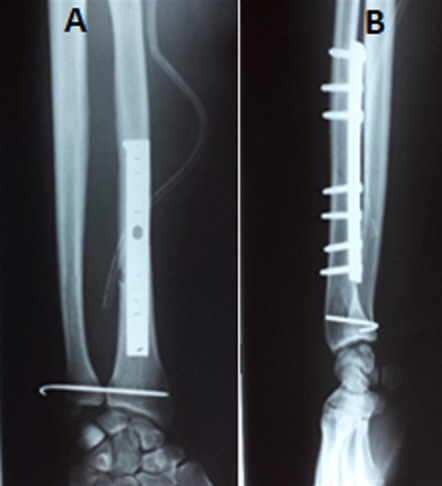
Ostéosynthèse par plaque vissée au niveau du radius et embrochage de l'articulation radio-ulnaire inférieur (A) face (B): profil

**Figure 5:**
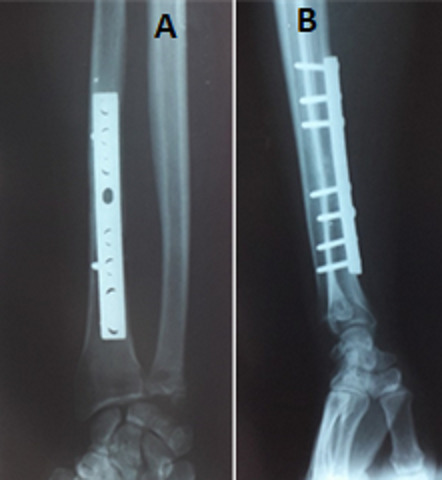
Radiographie des deux os de l'avant-bras prenant le poignet montrant la consolidation de la fracture (A) face; (B) profil

Les patients ont été opérés dans un délai moyen de 5,25 jours par plaque vissé dans 39 cas, par embrochage à ciel fermé dans 5 cas et par fixateur externe dans un cas. Dans 32 cas l'articulation RUD a été jugés stable et ont été immobilisés par plâtre. Dans 13 cas l'articulation RUD a été jugés instable, pour lesquelles on a eu recours à un embrochage transversal par broche sous contrôle d'amplificateur de brillance (Figure 4). L'ablation de la broche radio-ulnaire et des contentions plâtrées a été faite dans un délai moyen de 3 semaines (extrêmes de 21 à 60 jours). 38 patients ont adhéré au protocole de rééducation dès l'ablation de la broche et des contentions plâtrées. Le délai moyen de consolidation était de 8 semaines avec des extrêmes de 8 à 14 semaines (Figure 5). Tous nos patients ont été revus avec un recul moyen de 4 ans et 8 mois. Les résultats de notre série étaient les suivants: excellentes dans 35 cas (78%), bon dans 3 cas (7%), moyen dans 6 cas (13%) et mauvais dans un cas (2%). Dans notre série, le score de Mestdagh et al était excellent et bon pour les cas ayant une immobilisation courte, moyen et mauvais chez les patients qui n'ont pas eu de rééducation. Nous avons déploré 3 cas de sepsis sur broche ayant bien répondu aux soins locaux et au traitement antibiotique, 2 cas d'algodystrophie amélioré par le traitement médical et la rééducation et 2 cas de cal vicieux. Par ailleurs aucun cas de pseudarthrose ni de synostose radio-ulnaire n'a été noté au dernier.

## Discussion

La fracture luxation de Galeazzi est une lésion rare. Elle représente 2,7 à 6,8% de l'ensemble des fractures de l'avant-bras [1,3-6]. Dans notre série, la fracture luxation de Galeazzi représente 5,3% de l'ensemble des fractures de l'avant-bras (**Tableau 1**). Nous avons constaté que le siège de la fracture prédomine au niveau du le tiers distal de la diaphyse radial, ceci rejoint les données de la littérature [7-9]. Thomine [10] et Scott [11] pensent que l'atteinte de l'articulation RUD est pratiquement constante dans les fractures isolées de la diaphyse radiale, surtout si le trait de fracture siège au tiers distal ou à sa jonction avec le tiers moyen. Dans notre étude, selon la classification de Mansat et al, 89% des fractures ont été classé type II et III. La plupart des séries [7,12] rapportent la prédominance de type II et III, alors que le type I varie entre 0% et 6%. Nous avons obtenu 85% d'excellents et de bons résultats, 15% de moyens et mauvais résultats. Dansokho [13] a obtenu 86% d'excellents et de bons résultats, 14% de moyens et de mauvais résultats.

**Tableau 1:** Fréquence de la fracture luxation de Galeazzi

Auteurs	Nombre de fracture de Galeazzi	Nombre de fracture de l’avant-bras	Pourcentage (%)
GALEAZZI et al (1) (1934)	18	300	6
MOORE et al (3) (1985)	84	1236	6,8
PICHON et al (5) (2008)	13	383	4,8
KHALID et al (4) (2013)	28	647	4.3
KLOEN(6) (2010)	47	1120	4,3
Notre série	45	847	5,3

Rothit [14] et Pichon [5] retrouvent respectivement 93% et 89% d'excellents et de bons résultats. Dans notre série, pour les fractures de type I et II les résultats sont jugés excellents dans 90% des cas, pour les fractures de type III et IV dans 80% des cas. Dans certaines séries [13,7], les fractures de type I et II, les résultats étaient excellents dans 80 à 95% des cas. Tandis que pour les fractures de type III et IV le résultat n'est excellent que dans 50 à 60% des cas. Dans notre série, la prise en charge était dans les 48h du traumatisme dans 68,88% des cas. Ce délai varie selon les auteurs [4,15], il est de 1 à 30 jours. Dans notre série, le score de Mestdagh *et al*. était excellent et bon pour les cas ayant une immobilisation courte. Nous rejoignons la littérature [16], dans le résultat était jugé moyen à mauvais pour les patients immobilisés pendant une durée supérieure à 6 semaines. Dans notre série, nous avons adopté un protocole de rééducation bien codifié, 38 patients ont adhéré à ce protocole.

On rejoint Chirpaz et Mesplie qui recommandent la progressivité dans les mouvements de prono-supination d'abord passive et dès que le cal apparait devient actif et intensifié [17,18]. Nous avons constaté que le score fonctionnel de Mestdagh *et al.* des patients qui n'ont pas eu de rééducation était moyen et mauvais. Nous rejoignons les auteurs [2,19,20], qui ont eu des résultats jugés moyen à mauvais en raison d'une rééducation tardive et irrégulière ou complètement inadaptée. Dans notre série une douleur intermittente au niveau du poignet était rapportée chez 7 patients. La majorité des séries rapporte ces douleurs allant de douleurs permanentes, qui s'accentuent lors des mouvements de prono-supination, aux simples douleurs survenant uniquement à l'effort [7,21]. Le syndrome douloureux du poignet se manifeste aussi par une diminution de la mobilité portant sur la pronation et une diminution de la force de préhension. Les lésions ligamentaires de l'articulation RUD constituent la principale cause de cette instabilité pour Zlatkin [22], Michael [21], Lahrach [23] et Ruchelsman [24].

La pseudarthrose de la styloïde ulnaire, l'immobilisation prolongée de l'articulation RUD et la rééducation insuffisante constitue la cause de ces douleurs selon Budgen [25]. Nous n'avons pas noté de cas de pseudarthrose dans notre série, même pour le cas de fracture ouverte traité par fixateur externe. La pseudarthrose est retrouvé dans 2,7 à 3,5% selon les séries [3,26,27]. Deux cas de cal vicieux ont été notés dans notre série en angulation bien tolérée car la courbure pronatrice et la longueur du radius étaient peu modifiées. La littérature [25,] montre que ces cals vicieux sont devenus rares, et bien tolérés après ostéosynthèse par des plaques des fractures fraiches et déplacées du radius chez l'adulte. Ce sont les angulations de plus de 20° qui entraînent une diminution significative de la prono-supination. Une étude cadavérique a montré qu'en créant une désaxation de moins de 10° dans n'importe quel sens du radius a peu de retentissement sur la prono-supination [28]. Nous n'avons noté aucun cas de synostose radio-ulnaire. En effet, il s'agit d'une complication très rare dans la fracture luxation de Galeazzi [29].

## Conclusion

Au terme de notre travail et à la lumière des séries publiées, la fracture luxation de Galeazzi reste une lésion relativement fréquente en traumatologie du membre supérieur. Elle doit être suspectée systématiquement devant toute fracture isolée de la diaphyse radiale. Une ostéosynthèse solide et stable du foyer radial surtout par des plaques vissées permet d'obtenir la réduction de la radio-ulnaire distale et la consolidation osseuse dans les délais. La stabilisation de la radio-ulnaire distale par un embrochage distal horizontal ou par immobilisation plâtré est obligatoire et garant d'un bon résultant fonctionnel. L'immobilisation postopératoire est bénéfique permettant l'amélioration des délais de consolidation et de cicatrisation ligamentaire. La rééducation précoce permet d'avoir des bons résultats fonctionnels à court et à long terme.

### Etat des connaissances actuelles sur le sujet

- Les fractures de Galeazzi sont une entité assez fréquente et souvent sous diagnostiquée;
- Les fractures de Galeazzi posent un problème pronostique à cause des douleurs séquellaires au niveau du poignet;
- Le traitement des fractures de Galeazzi chez l'adulte est toujours chirurgical.

### Contribution de notre étude à la connaissance

- L'examen dynamique peropératoire après réduction solide est l'examen clé dans les fractures de Galeazzi;
- L'immobilisation courte du poignet donne de bons résultats fonctionnels;
- L'absence de rééducation post opératoire donne de mauvais résultats fonctionnels.

## Conflits d’intérêts

Les auteurs ne déclarent aucun conflit d'intérêts.
